# Impact of Titanium Dioxide Nanoparticles on Cd Phytotoxicity and Bioaccumulation in Rice (*Oryza sativa* L.)

**DOI:** 10.3390/ijerph17092979

**Published:** 2020-04-25

**Authors:** Wei Zhang, Jinghua Long, Jianmin Geng, Jie Li, Zhongyi Wei

**Affiliations:** 1 School of Public Administration, Hebei University of Economics and Business, Shijiazhuang 050061, China; zhangdaw007@heuet.edu.cn; 2 College of Mathematics and Statistics, Hebei University of Economics and Business, Shijiazhuang 050061, China; stgengjianmin@heuet.edu.cn; 3 College of Land and Environment, Shenyang Agricultural University, Shenyang 110866, China; lijie@syau.edu.cn (J.L.); weizy@syau.edu.cn (Z.W.)

**Keywords:** titanium dioxide nanoparticle, cadmium, bioaccumulation, rice

## Abstract

The impact of engineered nanoparticles (ENPs) on the migration and toxicity of coexisting pollutants is still unclear, especially in soil media. This study aims to evaluate the impact of titanium dioxide nanoparticles (TiO_2_ NPs) on the phytotoxicity of cadmium (Cd) to *Oryza sativa* L., and the migration of cadmium (Cd) in the soil-rice system. Three different Cd stress groups (C1 group: 1.0 mg kg^−1^, C2 group: 2.5 mg kg^−1^ and C3 group: 5.0 mg kg^−1^) were set in the pot experiment, and the target concentration of TiO_2_ NPs in each group were 0 mg kg^−1^ (T0), 50 mg kg^−1^ (T1), 100 mg kg^−1^ (T2) and 500 mg kg^−1^ (T3). Plant height and biomass decreased with the increasing of Cd content in paddy soil. TiO_2_ NPs could lower the phytotoxicity of Cd in terms of the changes in the morphological and biochemical characteristics, especially in the tillering and booting stage. In the tillering stage, TiO_2_ NPs addition caused a significant increase in plant height, biomass and the total chlorophyll content in the leaves of *Oryza saliva* L. In the booting stage, TiO_2_ NPs addition caused a 15% to 32% and 24% to 48% reduction of malondialdehyde (MDA) content for the C2 and C3 group, respectively, compared to that of the respective control treatment (T0). TiO_2_-NPs addition reduced the activity of peroxidase (POD) in the leaves in the booting and heading stage, and the activity of catalase (CAT) in the tillering stage. In the C1 and C2 group, the grain Cd content in the 100 and 500 mg kg^−1^ TiO_2_ NPs treatments reached 0.47–0.84 mg kg^−1^, obviously higher than that of the treatment without TiO_2_ NPs (0.27–0.32 mg kg^−1^), suggesting that TiO_2_-NPs could promote Cd migration in the soil-rice system.

## 1. Introduction

Nanomaterials have been widely used in industry, agriculture, medical and other fields in recent years, and a large number of nanoparticles can be discharged into the environment in the process of consume and disposal [1,2]. The environmental risk and biosafety of engineered nanoparticles have attracted the widespread attention of scientists and the public [3,4]. After entering the soil, engineered nanoparticles can affect soil microorganisms, plant growth, crop yield and food quality [5,6].

Due to their special surface properties, nanoparticles can influence the environmental behavior and toxicity of coexisting pollutants [7,8,9]. Titanium dioxide nanoparticles (TiO_2_ NPs) are commonly engineered nanoparticles (ENPs), that are widely used in cosmetics, plastics and coating, etc. Yang et al. (2012) found that cadmium (Cd) could be adsorbed by TiO_2_ NPs, and the TiO_2_ NPs additions could alleviate the toxicity of Cd to green alga *Chlamydomonas reinhardtii*. [10] TiO_2_ NPs could reduce the toxic effects of tetracycline on rice, in terms of its fresh biomass and antioxidant enzyme activities, and this could lead to a decrease in tetracycline content in the rice seedings [11]. Cai et al. (2017) found that 1000 mg L^−1^ TiO_2_ NPs exposure could reduce the Pb bioaccumulation in rice in a hydroponic experiment [12]. However, most of the experiments were conducted under hydroponic conditions; only a few experiments were conducted under soil conditions [13,14]. Chai et al. (2013) found that 2400 mg kg^−1^ carbon nanotubes (CNTs) improved Cd accumulation in shoots and reduced Cd accumulation in roots. CNTs alleviated Cd toxicity in terms of restoring shoot growth reduction, retrieving water content and resuming plant height [13]. Hu et al. (2014) found that graphene oxide could amplify the toxicity of arsenic to wheat, causing a decrease in biomass and root numbers [14].

In addition, most of the studies focused on the effect of nanoparticles on plants germination and seeding growth; few studies have explored the impact during the whole process of plant growth [15]. Thus, it is necessary to conduct further research on the effect of nanoparticles on the environmental behavior of coexisting pollutants in the soil system [16].

Cadmium pollution in paddy soil has become a serious environmental problem, especially in South China. The Cd in soil can be easily uptaken and enriched in rice grain, and influence the safety of agricultural products and human health [17,18]. Different materials, such as biochar, lime and hydroxyapatite, were used for Cd immobilization in soil [19]. In this study, different concentrations of TiO_2_ NPs were added to the Cd polluted paddy soils, and a pot experiment was conducted to (1) evaluate the impact of TiO_2_ NPs on the phytotoxicity of Cd to *Oryza sativa* L. during the whole process of plant growth; (2) investigate the influence of TiO_2_ NPs on the migration and bioaccumulation of Cd in the soil-rice system. This study could further improve our understanding on the environmental health risk of engineered nanoparticles.

## 2. Materials and Methods 

### 2.1. Soil and Nanoparticles Characteristics

The soil was sampled from a paddy field (0–30 cm) in Huizhou, Guangdong, South China (114°49′, 23°01′). Soil samples were air dried and passed through a 2 mm sieve. The content of soil organic matter was determined using a Multi N/C Total Organic Carbon Analyzer (Carl Zeiss, Jena, Germany). The cation exchange capacity was determined according to the ammonium acetate method. The content of organic matter was 2.45%, and the cation exchange capacity was 8.5 cmol kg^−1^ (Supporting Information (S1) Table S1). 

TiO_2_ NPs (Nanjing XFNANO Materials Tech Co., Ltd) properties were measured in our laboratory and reported in a previous study (Supporting Information (S1) Figure S1) [20]. The diameter size of TiO_2_ NPs ranges between 20–40 nm; the specific surface area is 77.4 m^2^ g^−1^.

### 2.2. Experimental Design

Three different Cd stress groups were set in this experiment, and the target concentrations of soil Cd in each group were 1.0 mg kg^−1^, 2.5 mg kg^−1^ and 5.0 mg kg^−1^ (C1 group, C2 group and C3 group). Different concentrations of CdCl_2_ solution were added to the soil samples in each different group, and mixed thoroughly in a plastic bucket. The Cd contaminated soils were equilibrated for three months, air dried, and passed through a 2 mm sieve. The final concentrations of soil Cd in different groups were 1.03 mg kg^−1^ (C1 group), 2.46 mg kg^−1^ (C2 group) and 5.06 mg kg^−1^ (C3 group). The main purpose of this study was to evaluate the impact of TiO_2_ NPs on Cd translocation and toxicity in the soil-rice system, thus, there was no No-Cd group.

TiO_2_ NPs powder was added into the Cd contaminated soil, and uniformly mixed. The target concentrations were 0 mg kg^−1^, 50 mg kg^−1^, 100 mg kg^−1^ and 500 mg kg^−1^ for TiO_2_-NPs. There was a total of 12 treatments, each with 3 replications (Table 1).

The soil-TiO_2_ NPs mixtures were placed into a nylon net bag (diameter 50 mm, height 100 mm, pore size 37 μm), and then the net bag was placed in the center of the plant pot (diameter 150 mm, height 300 mm) with 3 kg mixtures.

Rice (*Oryza sativa* L.) seeds (R7116) were provided by the Rice Research Institute, Guangdong Academy of Agricultural Science, Guangzhou, China. The details of seed culture and pot culture methods were described in previous studies [15,21]. Uniformed rice seedlings (height 80 mm) were transplanted to the center of the pot. The pot experiment was conducted in a greenhouse with natural light. Plants were irrigated with deionized water every day, to keep the water layer depth 3 cm above the soil surface. Then, 200 mg CO(NH_2_)_2_ kg^−1^ and 200 mg KH_2_PO_4_ kg^−1^ were added as fertilizers three times during the growing stage. The whole process of pot culture lasted for 4 months.

Plant height and tillering number were measured at 30, 60 and 90 days after transplanting, corresponding to tillering, booting and heading growth stages, respectively. After they were harvested, the roots, shoots and ears were collected separately, and washed and dried in a drying oven (40 °C) to a constant weight. The non-rhizosphere soil was sampled at the outside of the net bag (3 cm distance from the net bag). The root was taken from the net bag, and then shaken to remove large chunks of soil from the root; the rest of the soil that stuck to the root was determined to be rhizosphere soil. The rhizosphere soil was brushed down from the root with a small brush. Soil samples were collected from all treatments, air dried and passed through a 2 mm sieve.

### 2.3. Analysis of Cd Bioavailability

To determine the Cd content in soil and plant samples, soil samples were digested with the HCl-HNO_3_-HF-HClO_4_ mixed-acid digestion method, and plant samples (root, shoot and grain) were digested with the HNO_3_-HClO_4_ mixed-acid method [22]. In the current study, Cd contents determined in the root comprise both Cd uptake by the root (internalized Cd) and Cd adsorbed to the outer tissues of the root. The bioavailable Cd contents in the soil were extracted with 0.11 mL L^−1^ of HOAc [23]. The Cd concentrations in the extractant were measured using inductively coupled plasma mass spectrometry (Agilent 7700x ICP-MS). A certified soil reference material (GBW07430, National Research Center for Certified Reference Materials, China) was used to ensure the accuracy of the analytical data, and the accuracy ranged from 94.3% to 103.7%.

The bioavailability of heavy metal leads to their accumulation in the biota. The bioconcentration factor (BCF) is an important indicator for the evaluation of pollutant environmental behavior. The BCF was calculated by the following equation [1]:

(1) BCF=CplantCsoil

where $C_{plant}$ (mg kg^−1^) is the Cd content in the organs of *Oryza sativa* L., and $C_{soil}$ (mg kg^−1^) is the total Cd content in soil.

### 2.4. Analysis of Plant Biochemical Properties

The chlorophyll content, soluble protein content, malondialdehyde (MDA) content and antioxidant enzyme activities (superoxide dismutase (SOD), peroxidase (POD) and catalase (CAT)) in the leaves of *Oryza saliva* L. were measured on the 30th, 60th and 90th day after transplant. The Chlorophyll content was detected using a chlorophyll meter TYS-A (TOP, China). The activities of SOD, POD, CAT and the content of soluble protein and malondialdehyde (MDA) were analyzed using a spectrophotometer (TU-1901). The content of soluble protein was determined using Coomassie Brilliant Blue G-250. The content of malondialdehyde (MDA) was determined using the thiobarbituric acid (TBA)-based colorimetric method [24]. SOD activity was assayed by measuring the ability of the enzyme extract to inhibit the photochemical reduction of nitrotetrazolium blue chloride at 560 nm. After the enzyme extract reacted with H_2_O_2_, POD activity was measured based on the H_2_O_2_ decomposition rate, using guaiacol as a hydrogen donor, and CAT activity was estimated based on the decrease in absorbance at 240 nm [25].

### 2.5. Data Analysis

All the data were presented as mean ± standard deviation (SD) of the triplicates for each treatment, and analyzed using the SPSS software (IBM SPSS Statistics 20, Armonk, NY, USA). A one-way ANOVA followed by the Tukey-HSD test was used to analyze the differences among various groups. *p* < 0.05 indicated a significant difference. Different letters in the graph indicate significant difference (*p* < 0.05).

## 3. Results

### 3.1. Changes in Plant Morphological Characteristics

#### 3.1.1. Plant Height

**Figure 1:**
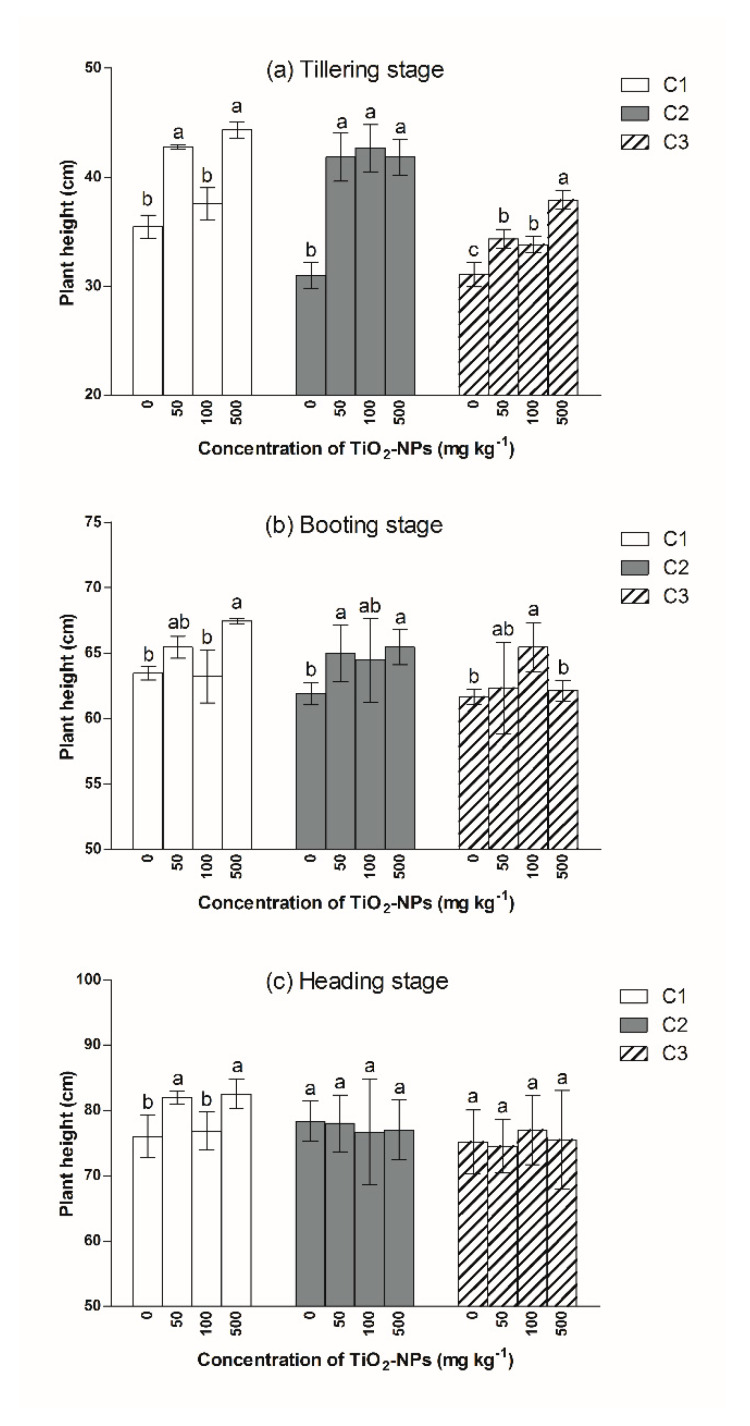
Plant height during various growth stages: (**a**) tillering stage, (**b**) booting stage, (**c**) heading stage. Different letters above column indicate significant difference (*p* < 0.05) between various treatment in same group.

Compared to the C1T0 treatment, exposure to Cd doses of 2.5 and 5.0 mg kg^−1^ had a noticeable (*p* < 0.05) impact on plant height, as there was a 13% and 12% decrease in the plant height for the C2T0 and C3T0 treatments, respectively (Figure 1a). The adverse effect of Cd on plant height increased with the Cd content in the tillering stage. In the C1 group, TiO_2_ NPs had a promoting effect on plant height in the tillering stage; plant height increased by 21%, 6% and 25% for 50, 100 and 500 mg kg^−1^ TiO_2_ NPs treatments, respectively, compared to the C1T0 treatment. In the C2 group, plant height increased by 35% to 42% across the TiO_2_ NPs treatments. There was no further improvement on plant height when the concentration of TiO_2_ NPs exceeded 50 mg kg^−1^ in the C1 and C2 group, while the opposite was observed in the C3 group. In the C3 group, the promoting effect of TiO_2_ NPs on plant height was enhanced, with an increase in the TiO_2_ NPs content, and the plant height in the 500 mg kg^−1^ TiO_2_ NPs treatment increased by 22% relative to the control treatment (C3T0). 

In the booting stage, no differences in plant height were observed between C1T0, C2T0 and C3T0 treatments (Figure 1b,c). In the C1 group, plant height in the C1T3 treatment was still significantly (*p* < 0.05) higher than that of the C1T0 treatment, while there was no clear difference between the C1T0, C1T1 and C1T2 treatments. In the C2 group, plant height increased by 3.1 cm and 3.5 cm for the C2T1 and C2T3 treatments, respectively, compared with the C2T0 treatment. Additionally, there was no clear difference between different TiO_2_ NPs content treatments. In the C3 group, plant height in the C3T2 treatment increased by 6% relative to that of the C3T0 treatment. 

In the heading stage, plant height increased by 8% and 9% for the C1T1 and C1T3 treatments, respectively, compared with C1T0 treatment (Figure 1c). However, in the C2 and C3 groups, there was no clear change in plant height across TiO_2_ NPs treatments when matched to respective control treatments.

#### 3.1.2. Rice Tillering

For every independent experiment group (C1, C2, and C3), no statistical difference in the tiller number or productive tiller number was observed between different TiO_2_ NPs content treatments (Supporting Information (S1) Table S2).

#### 3.1.3. Plant Biomass 

**Figure 2:**
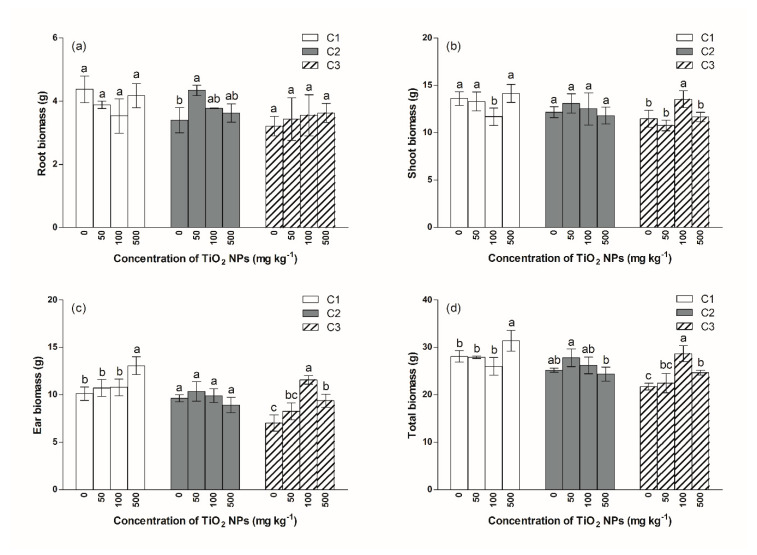
The impact of TiO_2_ NPs on plant biomass. (**a**) root biomass, (**b**) shoot biomass, (**c**) ear biomass, (**d**) total biomass. Different letters above column indicate significant differences (*p* < 0.05) between various treatment in same group.

Compared to the C1T0 treatment, exposure to Cd doses of 2.5 and 5.0 mg kg^−1^ had a noticeable (*p* < 0.05) impact on rice root biomass, as there was a 22% and 27% reduction in the root biomass of the C2T0 and C3T0 treatments, respectively (Figure 2a). In the C1, C2 and C3 groups, there were no significant differences between the root biomass seen in TiO_2_ NPs treatments and the matched control treatments, except in the C2T1 treatment, in which the root biomass increased by 28%, relative to the control (C2T0) treatment (Supporting information (S1) Table S3).

The impact of TiO_2_ NPs on shoot biomass was not clear. A decrease in shoot biomass was witnessed in the C1T1 treatment, while a slight increase in shoot biomass was witnessed in the C3T2 treatment (Figure 2b). 

As shown in Figure 2c, exposure to Cd clearly inhibited ear biomass; rice ear biomass decreased from 10.1 g in the C1T0 treatment to 7.0 g in the C3T0 treatment. In the C1 group, the ear biomass of the C1T3 treatment was significantly higher than that of the C1T0 treatment. In the C3 group, ear biomass in the C3T2 and C3T3 treatments was significantly (*p* < 0.05) higher than that of the C3T0 treatment.

TiO_2_ NPs had no clear impact on the total biomass in the C1 and C2 groups, except in the C1T3 treatment, where the total biomass increased by 12% relative to that of the C1T0 treatment (Figure 2d). In the C3 group, total biomass increased by 32% and 14% in the C2T2 and C3T3 treatments, respectively, relative to the control treatment.

### 3.2. Changes in Plant Biochemical Characteristics

#### 3.2.1. Chlorophyll Content in the Leaves of *Oryza saliva* L.

The addition of TiO_2_ NPs had an effect on the chlorophyll content in the leaves of *Oryza saliva* L. in the tillering stage (Supporting information (S1) Table S4). In the C1 group, the chlorophyll content in the leaves increased by 0.5, 0.3 and 0.9 mg kg^−1^ for the C1T1, C1T2 and C1T3 treatments, respectively, relative to the C1T0 treatment. In the C2 group, the chlorophyll content in the leaves increased by 0.3 and 0.4 mg kg^−1^ for the C2T2 and C2T3 treatments, respectively, relative to the C2T0 treatment. In the C3 group, the chlorophyll content in the leaves increased by 0.2 and 0.3 mg kg^−1^ for the C3T1 and C3T3 treatments, respectively, relative to the C3T0 treatment. However, the addition of TiO_2_ NPs had no significant impact on the chlorophyll content in the leaves in the booting and heading stage.

#### 3.2.2. Soluble Protein and MDA Content in the Leaves of *Oryza saliva* L.

**Figure 3:**
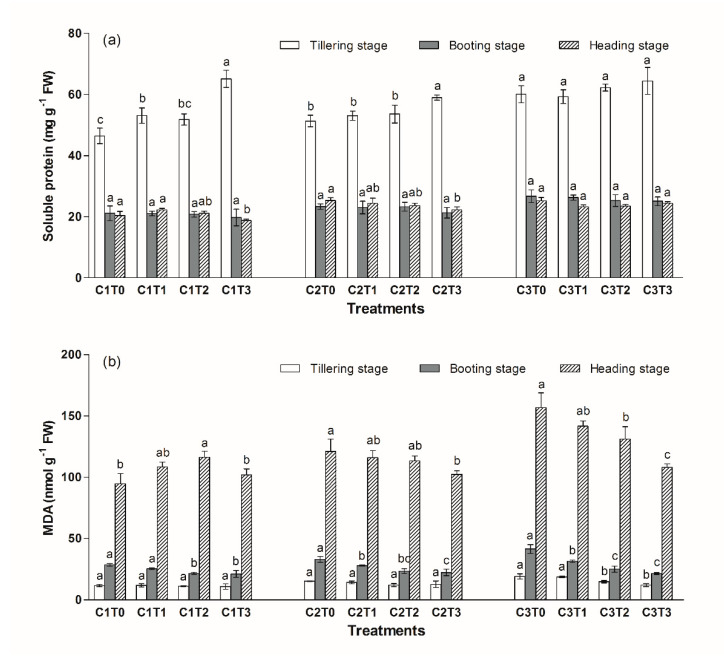
Changes in the soluble protein (**a**) and malondialdehyde (MDA) (**b**) in the leaves of *Oryza saliva* L. Different letters above columns indicate significant differences (*p* < 0.05) between various treatments in same growth stage.

In the tillering stage, compared to the C1T0 treatment, there was a 10% and 29% increase in the soluble protein content for the C2T0 and C3T0 treatments, respectively (Figure 3a). In the C1 group, the soluble protein content increased by 14% and 40% in the C1T1 and C1T3 treatments, respectively, relative to the C1T0 treatment. In the C2 group, the soluble protein content in the C2T3 treatment increased by 15%, compared with the C2T0 treatment in the tillering stage.

In the C1 group, the MDA content in the leaves of *Oryza saliva* L. decreased by 24% and 25% in the C1T2 and C1T3 treatment compared to that of the C1T0 treatment in the booting stage (Figure 3b). In the C2 and C3 group, the MDA content decreased with the increase of the TiO_2_-NPs content in soil. In the C2 group, the MDA content in the C2T1, C2T2 and C2T3 treatments decreased by 15%, 28% and 32%, respectively, relative to the C2T0 treatment in the booting stage. Furthermore, in the heading stage, the MDA content in the C2T3 treatment reduced by 15%, compared with the C2T0 treatment. 

In the C3 group, the MDA content in the leaves for the C3T2 and C3T3 treatments was significantly lower than that of the C3T0 treatment in the tillering stage. In the booting stage, the MDA content in the leaves for the C3T1, C3T2 and C3T3 treatments decreased by 24%, 40% and 48%, respectively, relative to the C3T0 treatment. In the heading stage, the MDA content in the C3T2, and C3T3 treatments decreased by 16% and 31%, respectively, relative to the C3T0 treatment.

#### 3.2.3. Antioxidant Enzyme Activities in the Leaves of *Oryza saliva* L.

**Figure 4:**
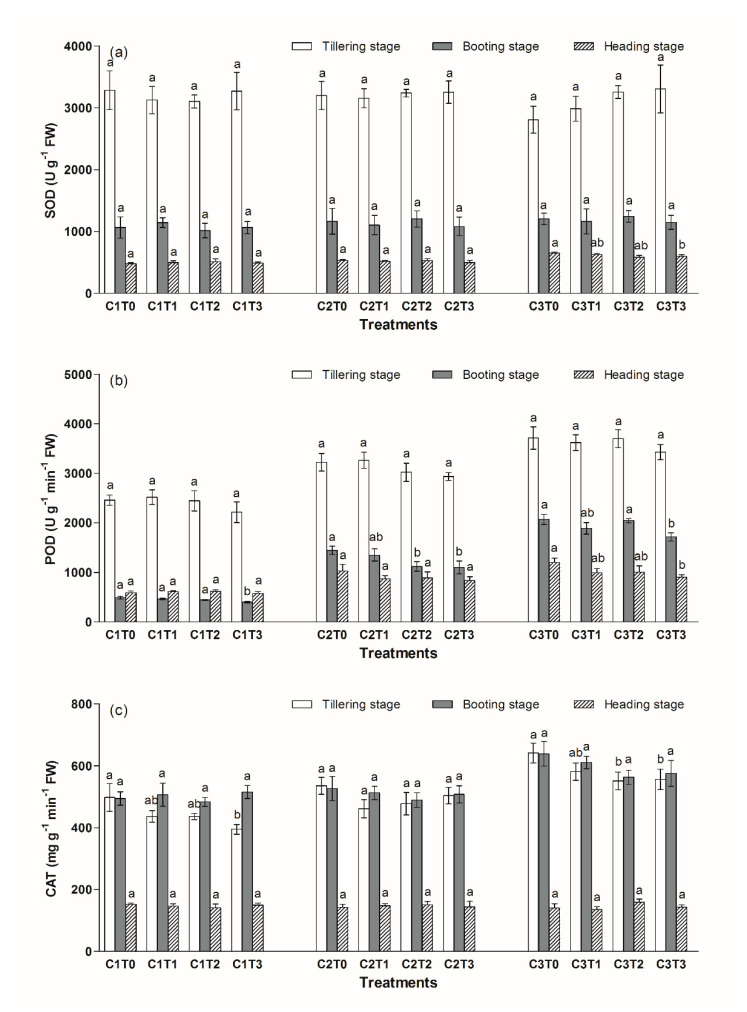
Changes in the superoxide dismutase (SOD) (**a**), peroxidase (POD) (**b**) and catalase (CAT) (**c**) activities in the leaves of *Oryza saliva* L. Different letters above columns indicate significant differences (*p* < 0.05) between various treatment in same growth stage.

Antioxidant enzyme activities can increase under adverse situations. The activities of POD and CAT in the leaves of *Oryza saliva* L. increased with the Cd content in the soil, while this increase was not observed for SOD (Figure 4). In the C1 and C2 group, the difference of SOD activities between different TiO_2_ NPs content treatments was not significant during the various growth stages (Figure 4a). In the C3 group, in the heading stage, the SOD activities in the C3T3 treatment reduced by 8% compared with the C3T0 treatment. 

Under different Cd stress conditions, TiO_2_ NPs significantly reduced the POD activities in the leaves, especially in the booting and heading stage (Figure 4b). In the C1 group, the POD activity in the C1T3 treatment decreased by 18% compared with the C1T0 treatment in the booting stage. In the C2 group, the POD activities in the C2T2 and C2T3 treatments reduced by 22% and 24%, respectively, compared with the C2T0 treatment in the booting stage. In the C3 group, the POD activity in the C3T3 treatment decreased by 17% compared with the C3T0 treatment in the booting stage, and a 25% reduction was also found in the heading stage.

In the tillering stage, TiO_2_ NPs significantly reduced the CAT activities in the leaves of *Oryza saliva* L. In the C1 group, the CAT activity in the C1T3 treatment decreased by 21% compared to that of the C1T0 treatment in the tillering stage (Figure 4c). In the C3 group, the CAT activities in the C3T2 and C3T3 treatments decreased by 14% and 13%, respectively, compared to that of the C3T0 treatment in the tillering stage. Meanwhile, this behavior was not observed for the C2 group.

### 3.3. Changes in Cd Bioavailability

#### 3.3.1. Bioavailable Cd Content in the Soil

The bioavailable Cd content in rhizosphere and non-rhizosphere soil was shown in Figure S2. In the C1 group, bioavailable Cd content in the non-rhizosphere soil decreased by 7% to 9% in the TiO_2_ NPs addition treatments, compared to that of the C1T0 treatment. In the C2 group, bioavailable Cd content in the non-rhizosphere soil decreased by 6% to 9% in the TiO_2_ NPs addition treatments compared to that of the C2T0 treatment, however, bioavailable Cd content in the rhizosphere soil increased by 2% to 6% with the TiO_2_ NPs addition. In the C3 group, there was no significant difference in Cd content between the rhizosphere soil and non-rhizosphere soil. TiO_2_-NPs caused a slight reduction of the bioavailable Cd content in the non-rhizosphere soil.

#### 3.3.2. Cd Content in Rice Plants

**Figure 5:**
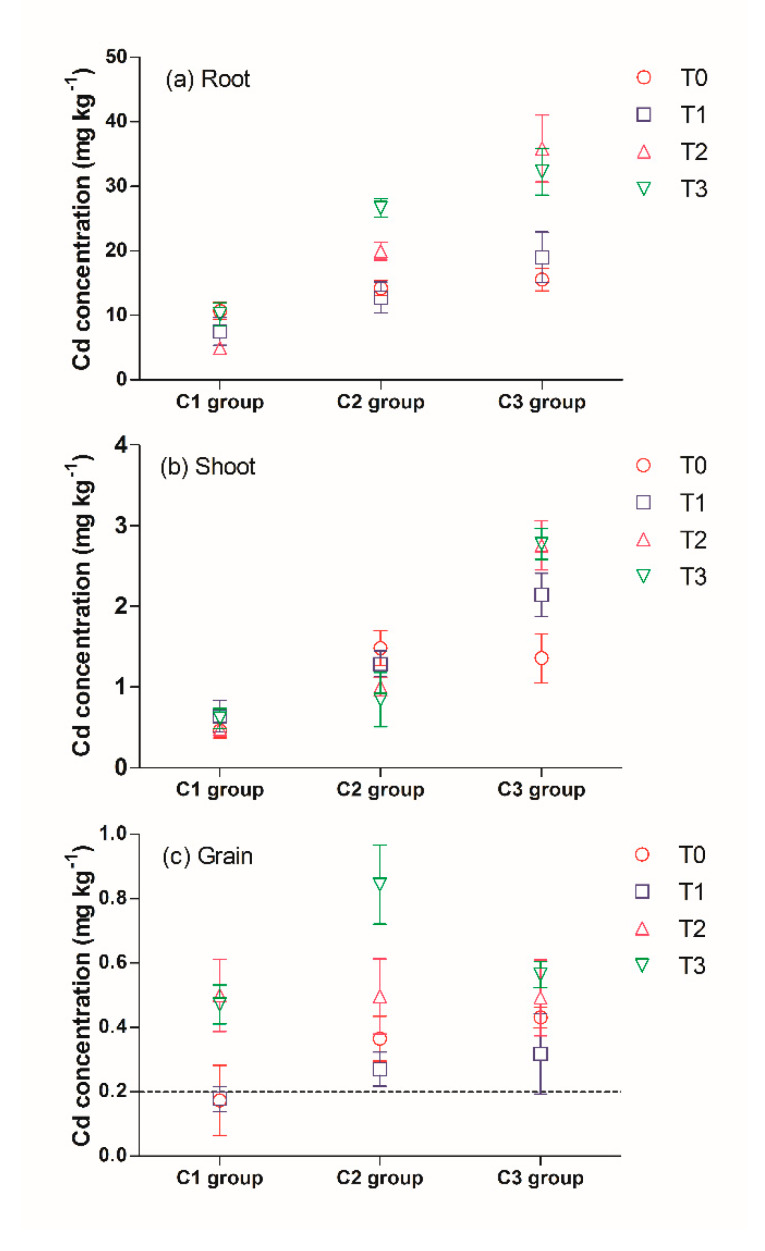
Cd content in different parts of plant: (**a**) roots, (**b**)shoots, (**c**) grain. ----: threshold of Cd content in rice grain, China (GB2762-2012).

The Cd content in the plant organs increased as the Cd content in the soil increased, and the Cd content in the root was highest, followed by shoot and grain (Figure 5).

In the C2 and C3 groups, the root Cd contents in the treatments with 100 and 500 mg kg^−1^ TiO_2_ NPs were significantly higher than that of the treatment without the TiO_2_ NPs addition (Figure 5a). In the C2 group, the root Cd content increased by 12.4 and 5.7 mg kg^−1^ for the C2T2 and C2T3 treatments, respectively, compared to that of the C2T0 treatment. In the C3 group, the root Cd content increased by 16.8 and 20.3 mg kg^−1^ for the C3T2 and C3T3 treatments, respectively, compared to that of the C3T0 treatment.

In the C3 group, TiO_2_ NPs addition caused a significant increase of Cd content in the shoot. The shoot Cd content increased by 0.78, 1.4 and 1.4 mg kg^−1^ for the C3T1, C3T2 and C3T3 treatments, respectively, relative to the C3T0 treatment (Figure 5b).

In the C2 and C3 group, 50 mg kg^−1^ TiO2 NPs caused a slight reduce of Cd content in the grain (Figure 5c). The grain Cd content in the C2T1 and C3T1 treatments decreased by 0.10 and 0.12 mg kg^−1^, respectively, compared to that of the respective control treatments, but still exceeded the threshold of Cd content in the rice grain of China (0.2 mg kg^−1^) (GB2762-2017). 

However, a high content (100 and 500 mg kg^−1^) TiO_2_ NPs addition significantly increased the Cd content in grain, especially in the C1 and C2 groups. In the C1 group, the grain Cd content in the C1T2 and C1T3 treatments increased to 0.50 and 0.47 mg kg^−1^, respectively, which is more than twice the amount allowed by China’s food safety limit. In addition, in the C2 group, the grain Cd content in the C2T2 and C2T3 treatments increased to 0.50 and 0.84 mg kg^−1^, respectively.

## 4. Discussion 

### 4.1. Effect of TiO_2_ NPs on Cd Toxicity

#### 4.1.1. Plant Morphological Characteristics

The study results showed that the plant height and biomass decreased with the increasing of Cd content in paddy soil; the phytotoxicity of Cd on *Oryza saliva* L. depended on the Cd content in soil. Compared to the control treatment (without TiO_2_ NPs), TiO_2_-NPs addition significantly accelerated the growth of *Oryza saliva* L. A significant increase of plant height and biomass was found in TiO_2_-NPs addition treatments, especially in the tillering and booting stages. The changes of plant morphological characteristics suggested that TiO_2_ NPs could lower the phytotoxicity of Cd. Under the flooding condition, Cd could be released from soil to the water, and then adsorbed by TiO_2_-NPs. TiO_2_-NPs could increase the adsorption capacity and reduce the mobility and toxicity of Cd [26]. Ji et al. (2017) also demonstrated that TiO_2_-NPs could promote the growth and development of the rice plant, and reduce the Cd content in plant organs, whereas their study was carried out in a hydroponic experiment, and only in the seedling stage [27]. However, TiO_2_-NPs had no significant impact on rice tillering, and there were no significant differences in plant height between different TiO_2_-NPs content treatments in the fruiting stage. Thus, the influencing mechanism of MNPs on plant growth, especially under the soil condition, is varied and deserves further investigation.

#### 4.1.2. Plant Biochemical Characteristics

Leaf chlorophyll content is an important indicator of photosynthetic capacity [28]. In this study, TiO_2_ NPs significantly increased the total chlorophyll content in the leaves of *Oryza saliva* L. in the tillering stage, which demonstrated that TiO_2_ NPs could enhance the photosynthetic efficiency of the plant and promote plant growth under Cd stress. Similarly, Servin et al. (2013) reported that 750 mg kg^−1^ TiO_2_ NPs addition increased the chlorophyll content in the leaves of *Cucumis sativus* L. [29].

As a product of lipid peroxidation, the MDA content can reflect the extent of plant senescence and resistance [30]. In this study, the MDA content in the leaves increased with the increasing of Cd content in soil. However, in the C2 and C3 groups, the MDA content decreased with the increasing of TiO_2_-NPs content, and showed that TiO_2_ NPs could inhibit the phytotoxicity of Cd.

In plants, exposure to Cd could not only affect photosynthesis, carbon and nitrogen metabolism, proteins synthesis, and antioxidant enzyme activities, but also generate reactive oxygen species (ROS) and subsequently lead to oxidative stress, all of which could affect plant growth [31,32,33]. Antioxidant enzyme activities can increase under adverse situations. As shown in Figure 4b,c, the activities of POD and CAT in the leaves of *Oryza saliva* L. increased with the Cd content in the soil. Under different Cd stress conditions, TiO_2_ NPs reduced the activity of POD in the leaves, especially in the booting and heading stage. In addition, a significant decrease in the CAT activities was observed in the tillering stage. The results indicated that TiO_2_ NPs inhibited the phytotoxicity of Cd to *Oryza saliva* L., causing an increase in chlorophyll content, and a decrease in antioxidant enzyme activities.

### 4.2. Effects of TiO_2_ NPs on Cd Bioaccumulation

**Figure 6:**
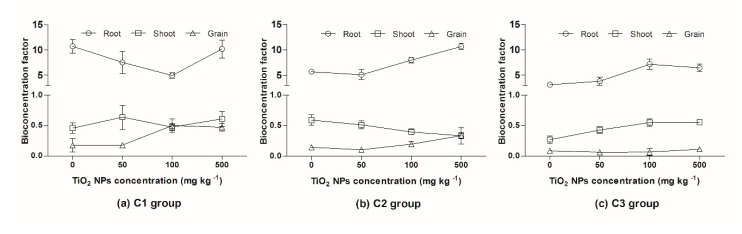
Changes in the Cd bioconcentration factor. (**a**) C1 group, (**b**) C2 group, (**c**) C3 group.

The Cd bioconcentration factor (BCF) of the root was highest, in the range of 3.1 to 10.7, and the BCF of shoot and grain were 0.3–0.7 and 0.1–0.5, respectively. The BCF of the root in the 500 mg kg^−1^ TiO_2_ NPs treatment was higher than in the 50 and 100 mg kg^−1^ treatments. In the C1 and C2 groups, the BCF of grain was higher in the 500 mg kg^−1^ TiO_2_ NPs treatment relative to the 50 and 100 mg kg^−1^ TiO_2_ NPs treatments (Figure 6). Cd was enriched in the rice root, and most of the Cd was retained in the root. In soil-rice system, Cd could be adsorbed on the surface of TiO_2_ NPs in the soil matrix and co-transport with TiO_2_ NPs, increasing the migration of Cd, and leading to an increased uptake of Cd by the root and increased translocation to the grain. 

These results suggested that TiO_2_ NPs could promote the Cd migration from soil to grain, especially in high TiO_2_ NPs content treatments. Similarly, Singh and Lee (2016) also found that the application of TiO_2_ NPs in soil could increase the Cd content in soybean plants under soil conditions [34]. However, Ji et al. (2017) found that TiO_2_ NPs reduced Cd accumulation in the rice roots and leaves under hydroponic condition [27]. The different results in the literature were caused by different experimental conditions and growth mediums [35]. The influencing mechanism of nanoparticles on the migration and bioavailability of co-contaminants under the soil condition still needs further investigation.

In addition, the total accumulation of Cd in the plant increased with the increasing concentrations of TiO_2_ NPs in soil. In the C2 group, the total accumulation of Cd in the 50, 100 and 500 mg kg^−1^ concentration TiO_2_ NPs treatments increased by 7%, 33% and 63%, respectively, compared to that of the control treatment. In the C3 group, the total accumulation of Cd in 50, 100 and 500 mg kg^−1^ concentration TiO_2_ NPs treatments increased by 33%, 149% and 126%, respectively. The root exudates could increase the solubility of Cd [34]. In this research, TiO_2_ NPs had a promote effect on rice plant growth; as a result, the increase of plant biomass led to the increase of the Cd accumulation in rice.

## 5. Conclusions

The present study investigated the impact of TiO_2_ NPs on Cd phytotoxicity and migration in the soil-rice system. On the basis of our observations, we draw the following conclusions.

The addition of TiO_2_ NPs in soil had an effect on the physiological parameters of *Oryza saliva* L., causing an increase in plant height, biomass and chlorophyll content, while causing a decrease in MDA content and antioxidant enzyme activities. Therefore, the presence of TiO_2_ NPs reduced the Cd phytotoxicity to *Oryza saliva* L.

However, TiO_2_-NPs addition did not reduce the Cd content in the grain to values below the maximum level set in the legislation. Therefore, the use of TiO_2_-NPs as a mitigation strategy to reduce the risk of Cd in the soil-rice system was infeasible, and the environmental behavior of nanoparticles in the soil-plant system, especially in the rhizosphere soil environment, deserves further investigation.

## Figures and Tables

**Table 1:** Experimental design.

Concentrations of Cadmium (Cd) (mg kg^−1^)	Concentrations of Titanium Dioxide Nanoparticles (TiO_2_ NPs) (mg kg^−1^)			
	0 (T0)	50 (T1)	100 (T2)	500 (T3)
1.0 (C1 group)	C1T0	C1T1	C1T2	C1T3
2.5 (C2 group)	C2T0	C2T1	C2T2	C2T3
5.0 (C3 group)	C3T0	C3T1	C3T2	C3T3

## Supplementary Materials

The following are available online at https://www.mdpi.com/1660-4601/17/9/2979/s1, Figure S1: Scanning electron microscope image of TiO_2_ NPs. Figure S2: Bioavailable Cd content in rhizosphere/non-rhizosphere soil. Table S1: Basic physicochemical properties of paddy soils, Table S2: Plant height during various growth stages, Table S3: The impact of TiO_2_ NPs on plant biomass, Table S4: Total chlorophyll content in the leaves of *Oryza saliva* L.
