# Supplementary material for: Impact of Titanium Dioxide Nanoparticles on Cd Phytotoxicity and Bioaccumulation in Rice (Oryza sativa L.)
Source: Int J Environ Res Public Health. 2020 Apr 25;17(9):2979. doi: 10.3390/ijerph17092979 (PMC7246507; doi:10.3390/ijerph17092979)
Supplement: Supplementary file 1 [file ijerph-17-02979-s001.pdf]

# Supplementary Data

# Impact of titanium dioxide nanoparticles on Cd phytotoxicity and bioaccumulation in rice (*Oryza sativa* L.)

**Table S1.** Basic physicochemical properties of paddy soils.

| Test Items | Paddy Soil |
|---|---|
| pH | 6.2 ± 0.1 |
| Organic matter (%) | 2.5 ± 0.1 |
| Sand fraction (%) | 14 ± 0.9 |
| Silt fraction (%) | 56 ± 0.5 |
| Clay fraction (%) | 31 ± 0.4 |
| Cation exchange capacity (cmol $kg^{-1}$) | 8.5 ± 0.1 |
| Total N (g $kg^{-1}$) | 2.3 ± 0.7 |
| Total P (g $kg^{-1}$) | 0.5 ± 0.1 |
| Total K (g $kg^{-1}$) | 12 ± 3.7 |
| Total Fe (g $kg^{-1}$) | 9.4 ± 0.7 |
| Total Mn (mg $kg^{-1}$) | 93 ± 8.1 |
| Total Cu (mg $kg^{-1}$) | 23 ± 3.2 |
| Total Zn (mg $kg^{-1}$) | 56.74 ± 3.19 |
| Total Cd (mg $kg^{-1}$) | 0.20 ± 0.04 |

**Table S2.** Plant height during various growth stages (cm).

| Treatment | Tillering Stage | | | Booting Stage | | | Heading Stage | | |
|---|---|---|---|---|---|---|---|---|---|
| | C1 Group | C2 Group | C3 Group | C1 Group | C2 Group | C3 Group | C1 Group | C2 Group | C3 Group |
| T0 | 35 ± 1.1 b | 31 ± 1.2 b | 31 ± 1.1 c | 63 ± 0.5 ab | 62 ± 0.8 b | 62 ± 0.6 b | 76 ± 3.3 b | 78 ± 3.1 a | 75 ± 4.9 a |
| T1 | 43 ± 0.2 a | 42 ± 2.2 a | 34 ± 0.9 b | 65 ± 0.8 ab | 65 ± 2.2 a | 62 ± 3.5 ab | 82 ± 1.0 a | 78 ± 4.4 a | 75 ± 4.1 a |
| T2 | 38 ± 1.5 b | 43 ± 2.2 a | 34 ± 0.7 b | 63 ± 2.0 b | 64 ± 3.2 ab | 65 ± 1.9 a | 77 ± 2.9 b | 77 ± 8.1 a | 77 ± 5.3 a |
| T3 | 44 ± 0.7 a | 42 ± 1.6 a | 38 ± 0.8 a | 67 ± 0.2 a | 65 ± 1.3 a | 62 ± 0.8 b | 83 ± 2.3 a | 77 ± 4.6 a | 76 ± 7.6 a |

Note: Different letters represent significant differences between various treatments in same group ($p < 0.05$, LSD), $n = 3$.

**Table S3.** The impact of $TiO_2$ NPs on plant biomass (g).

| Treatment | Root Biomass | | | Shoot Biomass | | | Ear Biomass | | | Total Biomass | | |
|---|---|---|---|---|---|---|---|---|---|---|---|---|
| | C1 Group | C2 Group | C3 Group | C1 Group | C2 Group | C3 Group | C1 Group | C2 Group | C3 Group | C1 Group | C2 Group | C3 Group |
| T0 | 4.3 ± 0.4 a | 3.4 ± 0.4 b | 3.2 ± 0.3 a | 14 ± 0.7 a | 12 ± 0.6 a | 11 ± 0.9 b | 10 ± 0.7 b | 9.6 ± 0.6 a | 7.0 ± 0.9 a | 28 ± 1.2 b | 25 ± 0.4 ab | 22 ± 0.7 c |
| T1 | 3.9 ± 0.1 a | 4.3 ± 0.2 a | 3.4 ± 0.7 a | 13 ± 1.0 a | 13 ± 1.0 a | 11 ± 0.6 b | 11 ± 0.9 b | 10 ± 1.0 a | 8.3 ± 0.9 bc | 28 ± 0.2 b | 28 ± 1.9 a | 22 ± 2.0 bc |
| T2 | 3.5 ± 0.5 a | 3.8 ± 0.1 ab | 3.6 ± 0.6 a | 12 ± 0.9 b | 12 ± 1.7 a | 14 ± 0.9 a | 11 ± 0.9 b | 9.9 ± 0.7 a | 12 ± 0.5 a | 26 ± 1.9 b | 26 ± 1.8 ab | 29 ± 1.7 a |
| T3 | 4.2 ± 047 a | 3.6 ± 0.3 ab | 3.6 ± 0.3 a | 14 ± 0.9 a | 14 ± 0.9 a | 12 ± 0.5 b | 13 ± 0.9 a | 8.9 ± 0.8 a | 9.4 ± 0.7 b | 31 ± 2.2 a | 24 ± 1.5 b | 25 ± 0.5 b |

Note: Different letters represent significant differences between various treatments in same group ($p < 0.05$, LSD), $n = 3$.

**Table S4.** Total chlorophyll content in the leaves of *Oryza saliva* L. (mg $kg^{-1}$).

| Treatment | Tillering Stage | | | Booting Stage | | | Heading Stage | | |
|---|---|---|---|---|---|---|---|---|---|
| | C1 Group | C2 Group | C3 Group | C1 Group | C2 Group | C3 Group | C1 Group | C2 Group | C3 Group |
| T0 | 4.2 ± 0.1 a | 4.5 ± 0.1 a | 4.4 ± 0.1 a | 3.8 ± 0.1 a | 4.0 ± 0.2 a | 4.3 ± 0.1 a | 3.1 ± 0.1 a | 3.1 ± 0.1 a | 3.1 ± 0.2 a |
| T1 | 4.6 ± 0.1 b | 4.7 ± 0.1 ab | 4.6 ± 0.1 b | 3.9 ± 0.1 a | 4.1 ± 0.1 a | 4.1 ± 0.2 a | 3.1 ± 0.2 a | 3.0 ± 0.3 a | 3.1 ± 0.1 a |
| T2 | 4.5 ± 0.1 b | 4.9 ± 0.1 bc | 4.5 ± 0.1 ab | 4.0 ± 0.1 a | 4.1 ± 0.2 a | 4.1 ± 0.1 a | 3.0 ± 0.1 a | 3.1 ± 0.1 a | 3.0 ± 0.2 a |
| T3 | 5.1 ± 0.1 c | 5.0 ± 0.1 c | 4.7 ± 0.1 b | 3.9 ± 0.2 a | 3.9 ± 0.1 a | 4.1 ± 0.2 a | 3.1 ± 0.2 a | 3.0 ± 0.2 a | 3.1 ± 0.2 a |

Note: Different letters represent significant differences between various treatments in same group ($p < 0.05$, LSD), n = 3.

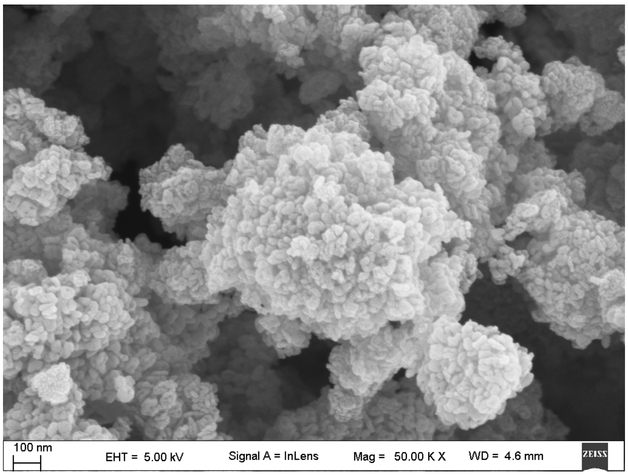


**Figure S1.** Scanning electron microscope image of $TiO_2$ NPs.

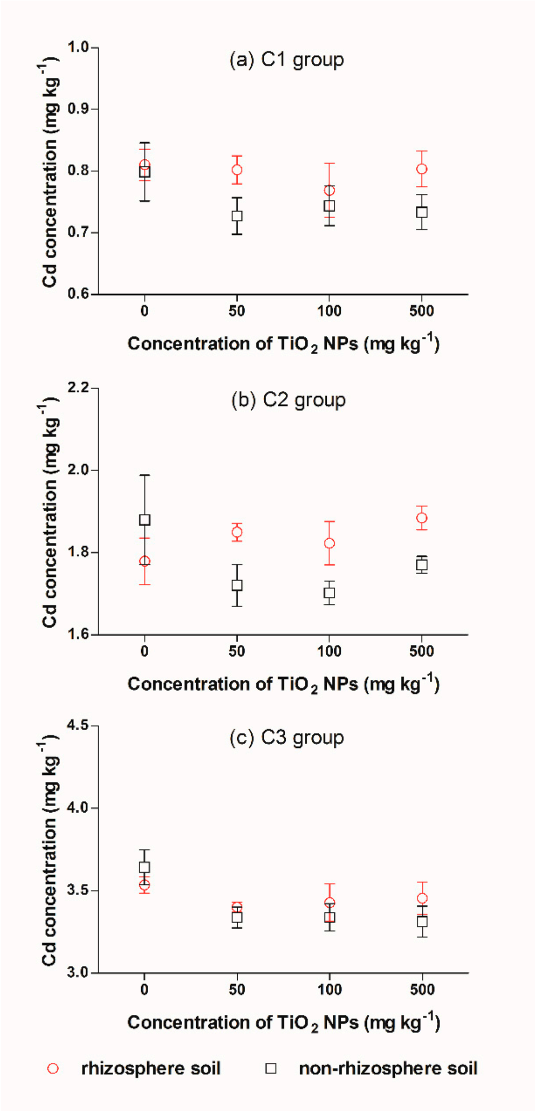


**Figure S2.** Bioavailable Cd content in rhizosphere/non-rhizosphere soil. (**a**) C1 group, (**b**) C2 group, (**c**) C3 group.
